# Weathering the Pain: Ambient Temperature’s Role in Chronic Pain Syndromes

**DOI:** 10.1007/s11916-025-01361-8

**Published:** 2025-01-23

**Authors:** David S. Jevotovsky, Whitman Oehlermarx, Tommy Chen, Christopher Chiodo Ortiz, Annie Liu, Sidharth Sahni, Jason L. Kessler, Joseph J. Poli, Richard Lau

**Affiliations:** 1 Department of Physical Medicine & Rehabilitation, New York University Langone Health, New York, NY USA; 2 Touro College of Osteopathic Medicine, Middletown, NY, USA; 3 Department of Anesthesia and Perioperative Care, University of California San Francisco Medical Center, San Francisco, CA USA; 4 Department of Anesthesiology and Perioperative Medicine, University of Rochester Medical Center, Rochester, NY USA

**Keywords:** Ambient temperature, Chronic pain, Fibromyalgia, Multiple sclerosis, Complex regional pain syndrome, Osteoartritis

## Abstract

**Purpose of Review:**

Chronic pain is highly prevalent and involves a complex interaction of sensory, emotional, and cognitive processes, significantly influenced by ambient temperature. Despite advances in pain management, many patients continue to experience inadequate pain relief. This review aims to consolidate and critically evaluate the current evidence on the impact of ambient temperature on chronic pain conditions such as fibromyalgia (FM), multiple sclerosis (MS), complex regional pain syndrome (CRPS), and osteoarthritis (OA).

**Recent Findings:**

Patients with FM often report pain exacerbations due to temperature changes, with studies showing lower thresholds for heat and cold-induced pain compared to healthy controls. In MS, the Uhthoff phenomenon, characterized by temperature-induced neurological deterioration, underscores the significance of ambient temperature in pain management. CRPS patients exhibit heightened pain sensitivity to temperature changes, with both warm and cold stimuli potentially aggravating symptoms. OA patients frequently report increased pain and rigidity associated with lower temperatures and higher humidity.

**Summary:**

Understanding the mechanisms through which temperature influences pain can enhance pain management strategies. This review highlights the need for further research to elucidate these mechanisms and develop targeted interventions, ultimately improving the quality of life for individuals with chronic pain conditions.

## Introduction

Pain is mediated by an intricate interplay of sensory, emotional, and cognitive processes. It is a universal human experience influenced by a multitude of factors, among which ambient temperature plays a crucial role. Chronic pain is a prevalent and challenging condition in the United States, affecting approximately 50 million adults (around 20% of the US population) and imposing substantial economic costs estimated at over $600 billion annually [1, 2]. Despite advances in pain management, many individuals with chronic pain experience inadequate pain relief and impaired quality of life.

One intriguing aspect of pain perception is its apparent susceptibility to environmental changes, including variations in temperature and weather patterns. Patients with chronic pain often report that their symptoms fluctuate with changes in temperature, humidity, and atmospheric pressure. Cold weather is commonly cited as a trigger for increased pain [3–6]. While such observations have long been considered anecdotal, emerging research has begun to elucidate the mechanisms underlying these phenomena.

Studies investigating the relationship between ambient temperature and pain have suggested that changes in temperature can influence the release of endogenous opioids, such as beta-endorphins, which play a crucial role in pain modulation [4]. VR1, a member of the TRPV group of transient receptor potential ion channels located in the dorsal roots of primary afferent nerves, has also been implicated in the relationship between temperature and pain [7]. However, the precise mechanisms through which temperature influences pain processing are still not fully understood, and the existing literature is inconsistent regarding the strength and direction of these associations.

The relationship between ambient temperature and pain is complex, particularly in chronic pain conditions, highlighting a need for a comprehensive review to consolidate and critically evaluate the existing evidence. This review aims to fill this gap by exploring the impact of ambient temperature on pain in several common chronic pain conditions, including fibromyalgia, complex regional pain syndrome, multiple sclerosis, and osteoarthritis. By synthesizing the current research findings, we seek to provide clarity regarding the influence of ambient temperature on pain perception. We hope this knowledge can be translated into more effective pain management strategies for patients with these conditions.

## Evidence by Clinical Condition

### Fibromyalgia

Fibromyalgia (FM) is a debilitating pain condition with uncertain etiology. With a prevalence of 6.4% in the United States, fibromyalgia affects women (7.7%) disproportionately more than men (4.9%) [8]. The poor understanding of underlying pathophysiology limits therapeutic options to symptomatic management with modalities such as cognitive behavioral therapy, exercise therapies and/or medications that target neuropathic pain [9].

Current research suggests patients with FM have an altered perception of pain. The high prevalence of peripheral small fiber nerve pathology in these patients, involving blood flow disturbance, reduced intraepidermal nerve fiber density, variant transient receptor potential ankyrin 1 (TRPA1), and variant catechol-O-methyltransferase (COMT) gene, may contribute to FM pain [10–14]. Variants in TRPA1 were found in individuals with FM who reported extreme pain exacerbated by the cold (11. Similarly, variants in COMT genes were also noted in FM with decreased cold sensitivity pain thresholds [14].

Irrespective of the uncertain pathophysiology of FM pain, patients often report exacerbations triggered by changes in weather conditions and ambient temperature [15, 16]. Both cold and warm/hot temperatures may influence symptoms [15–18]. Interest in the relationship between rheumatic diseases and meteorological variables has persisted for almost a century. The term “weather sensitive” was adopted to describe individuals who complained of pain due to changes in weather or temperature [17]. In 1994, Haggland et al. examined the relationship between actual weather, disease severity, and symptom reports for participants with FM. They found no correlation between myalgic scores nor tender point index scores in FM participants with respect to temperature, concluding that there are no physiologic changes associated with the weather [17]. However, this study did not address the relationship of higher incidence of pain reporting associated with changes in ambient temperature. A more recent study conducted in 2020 by Ten Brink et al. also found that both cold and warm/hot ambient temperatures act as intensifiers and triggers for pain, discomfort, and distress when compared with pain-free control [15].

Due to conflicting reports on the relationship between ambient temperature and FM pain, quantitative sensory testing has been conducted to better understand this relationship. Hot or cold sensitivity in FM patients using thermode stimuli is particularly well-described. Hurtig et al. found that heat and cold-related pain thresholds differed significantly between healthy controls and FM participants. FM individuals experienced heat pain at an average 41.1 °C (standard deviation [SD] 3.3) compared to the 45.2 °C (SD 3.3) threshold experienced by healthy controls. The cold-related pain threshold of FM individuals was at an average temperature of 19.7 °C (SD 6.7) compared to healthy participants who experienced cold-related pain at an average temperature of 8.4 °C (SD 3.7) [19]. The 4.1 and 11.3 °C difference in heat- and cold-related pain thresholds, respectively, highlight the influence of temperature on FM pain. A more recent systematic review on fibromyalgia pain corroborates previous literature, reinforcing the idea that FM individuals experience a lower threshold towards cold and hot temperature with more significant temperature differences between healthy controls [16]. This review found that FM individuals perceived cold-related pain between 10.9 and 26.3 °C, compared to the 5.9 to 13.5 °C range perceived in healthy controls [16]. Studies also demonstrate that exposure to longer periods of extreme temperature may not lead to habituation for the FM individuals compared to healthy individuals [18].

The intricate relationship between FM and temperature-induced pain underscores the complexity of this condition’s pathophysiology. As FM patients often report exacerbation of symptoms in response to weather changes, understanding these temperature-related pain mechanisms is crucial for tailored management approaches. Despite advances in our comprehension, much remains to be elucidated regarding how FM patients uniquely respond to pain stimuli across different temperature ranges. Therefore, further research into the nuanced interplay between FM and temperature sensitivity is imperative to inform effective strategies for managing FM-related pain and improving the quality of life for those affected by this complex condition.

### Multiple Sclerosis - Uhthoff Phenomenon

The Uhthoff phenomenon was first described by Wilhelm Uhthoff, a German ophthalmologist, who noted exercise-induced amblyopia in patients with multiple sclerosis (MS) [20]. Further observations led to the realization that heat was the nidus and that a wide array of neurologic symptoms such as increased pain could present themselves under the Uhthoff phenomenon. The correlation between this phenomenon and MS is so high that in the 1950s the diagnosis of MS was made on the results of the hot bath test [21]. This has led to our modern-day definition of the Uhthoff phenomenon as paroxysmal (typically lasting less than 24 h) neurologic deterioration induced by increased core body temperature. We now know that the Uhthoff phenomenon can be seen in up to 80% of western patients with MS [22].

The pathophysiology of the Uhthoff phenomenon is incompletely understood but thought to be related to the temperature-dependent central motor conduction block of partially demyelinated axons [23]. Demyelinated axons lead to unmasking of potassium channels and current leak, resulting in hyperpolarization. As an adaptive mechanism, new sodium channels are inserted into the axonal membrane. These new channels have altered physiology, and it has been shown that temperature escalations of as little as 0.2 to 0.5 °C is enough to enact temperature-induced pore closure and termination of depolarization [24]. Increased core body temperature is the underlying mechanism, however, the Uhthoff phenomenon has also been described in the setting of fever, sun-tanning, hot showers, sauna use, physiological stress, hot meals, and smoking cigarettes [25].

While the Uhthoff phenomenon was first described in the setting of optic manifestations, neurogenic deterioration and exacerbations of MS can manifest in a wide array of symptoms such as chronic pain and generally can be categorized as motor, sensory, or cognitive. A relatively common motor symptom is spasticity, which occurs in more than 80% of individuals with MS, and can produce involuntary, stiff and painful muscle movements [26]. Further, up to 90% of multiple sclerosis patients report central or musculoskeletal pain and is associated with the development of plaques and lesions along the spinothalamic tract [27]. Roughly 50% of patients with MS experience pain and altered sensation, with 8% of patients experiencing mechanical allodynia and others experiencing dysesthesia or Lhermitte’s phenomenon (electric shock sensation with neck flexion) [27]. It has been shown that increases in core temperature by even 0.5 °C can trigger neurologic deterioration and generally lasts until the core temperature returns to baseline [27]. One survey reported that, among patients with MS and heat sensitivity, the majority preferred a room temperature of less than 20 °C [28].

Treatment of the Uhthoff phenomenon includes body surface cooling and oral 4-aminopyridine. 4-aminopyridine, an inhibitor of potassium channels, prolongs the action potential and mitigates the effects of temperature-induced pore closure of sodium channels [29]. Future research is needed to better understand the physiologic mechanism behind the Uhthoff phenomenon, and better quantify the array of sensory and motor symptoms associated with this phenomenon in order to prevent deleterious effects on MS pain and function.

### Complex Regional Pain Syndrome

Complex regional pain syndrome (CRPS) is a chronic pain condition that typically includes hyperalgesia and allodynia [30]. Though CRPS subtypes are distinguished by the presence (Type I) or absence (Type II) of a nerve injury, both subtypes typically follow some form of trauma [31]. Reports in the US indicate a rate of 5.46 cases per 100,000 person-years at risk, while the Netherlands reports 26.2 cases per 100,000 person-years at risk, with a higher incidence among females aged 61–70 years old [32, 33]. Given the high prevalence of this debilitating condition, these statistics underscore the urgent need to study complex regional pain syndrome in order to develop more effective treatments.

Recent research into the pathophysiology of CRPS highlights the interplay between dysregulated inflammation, the autonomic nervous system, and autoimmunity [34]. Evidence also supports that hyperalgesia and allodynia in CRPS may be attributed at least in part to the activation of keratinocyte by Substance P and CGRP [35], as well as degradation of A-a nerve fibers with subsequent compensatory activation of A-delta fibers [36]. CRPS patients may have greater numbers of alpha-1 adrenergic receptors on keratinocytes and nociceptors, which can be linked to clinical autonomic components of pain [37, 38]. Autoimmunity also plays a role in CRPS pathophysiology, with evidence suggesting that autoantibodies sensitize nociceptors through alpha-1 adrenergic, beta-2 adrenergic, and M2 muscarinic receptors, resulting in the perception of pain [39–41].

In Type II CRPS, it has been postulated that abnormalities in skin blood flow, within the territory of the lesioned nerve, are caused by peripheral impairment of sympathetic function and sympathetic denervation [38, 42]. During the first weeks after transection of vasoconstrictor fibers, vasodilatation is present in the affected area. Later, the vasculature may develop an increased sensitivity that amplifies the response to local cold-temperature stimuli and also to catecholamines, presumably because of upregulation of adrenoceptors [42].

The International Association for the Study of Pain (IASP)’s Budapest criteria defines CRPS as pain that is disproportionate to any known injury with 2 or more signs in at least 3 of the 4 categories: sensory, vasomotor, sudomotor/edema, and motor/trophic; these signs include alterations in skin temperature [43]. There is a significant body of research exploring temperature thresholds (heat and cold allodynia) as well as the pathophysiology surrounding alteration in skin temperature in CRPS. However, the effect of ambient temperature remains largely unexplored.

Given that pain and temperature changes are a key presentation of CRPS, further exploring the role of ambient temperature may be important for understanding fluctuations in these patients’ pain. A more recent online survey conducted by Ten Brink et al. explored the role of ambient temperature on pain in patients with CRPS. They compared responses between CRPS, FM, pain controls, and pain-free controls. Individuals with CRPS more often reported that warm/hot weather triggered or intensified pain. Further, they found lower thresholds for warm/hot weather to trigger pain for patients with CRPS or FM versus pain free controls [15].

In a case study by Wasner et al. they describe a patient with CRPS I, with symptoms in the right upper extremity, and suggest that persistent vasodilation of the affected area may contribute to the patient’s pain [44]. Using a whole-body thermal suit on the patient to simulate changes in ambient temperature, the researchers were able to alter the patient’s perceived pain; warming and orthostatic load intensified the pain, whereas cooling and elevation provided pain relief [44]. The patient’s pain symptoms eventually reversed over the course of several weeks alongside overall clinical improvement and recovery [44]. Further research has additionally supported the idea that vasoconstriction may affect CRPS pain in the chronic phase [45]. These findings reinforce temperature’s inherent link to CRPS pain and suggest possible targets for future intervention.

The current literature suggests ambient temperature may be a catalyst for increased pain in CRPS patients. The limited data presented also suggests that ambient temperature may play a significant role in pain exacerbation in CRPS. More research is needed to better elucidate the role ambient temperature plays in the symptomatology of CRPS in order to provide clear guidance for patients and providers on symptom management.

### Osteoarthritis

Osteoarthritis (OA) is characterized by progressive degeneration of articular cartilage and remains one of the most prevalent chronic conditions, with more than 500 million people suffering globally [46]. OA poses an incredible burden on healthcare and subsequent economic impact, necessitating approximately $400 million dollars in annual direct and indirect spending in the United States alone [47].

Pain perception in OA is known to be impacted by temperature, with many patients endorsing increased pain with changes in ambient temperature, especially in cold and damp conditions [48]. It is a long-standing adage that weather influences individuals’ symptoms of osteoarthritis. One small study of 25 individuals sought to establish the root cause of this and explored the relationship between the pain and rigidity of arthritis and the weather variables of temperature, relative humidity, barometric pressure, wind speed, and precipitation. They found that lower temperature and higher relative humidity are associated with increased pain and rigidity [49].

McAlindon et al. (2007) examined the effects of weather conditions on pain and physical function in patients with symptomatic knee osteoarthritis. They noted colder temperatures were significantly associated with exacerbation of pain and reduced mobility [47]. These findings suggest that weather conditions play a significant role in OA pain and function, and may inform daily health management practices for individuals with OA.

More recently, Wang, et al. conducted a recent qualitative systematic review, which included 14 studies, and found that any meteorological condition was associated with OA pain. Subsequently, a quantitative meta-analysis was conducted to explore the association between weather conditions and osteoarthritis (OA) pain. They found strong evidence suggesting that weather factors, including temperature (T), barometric pressure (BP), and relative humidity (RH), were associated with OA pain. Specifically, BP and RH were positively correlated with OA pain intensity, while T showed a negative correlation. The meta-analysis revealed moderate correlations between OA pain and T or BP, and a weak correlation with RH. Despite the subjective nature of pain assessment and variability in study designs, the findings suggest that weather conditions strongly influence OA pain [50].

Fu et al. assessed the relationship between weather factors and pain exacerbations in individuals with symptomatic hip osteoarthritis (OA). A significant association was found between greater average daily temperature variation over 72 h and an increased risk of hip pain exacerbations [51]. However, no significant associations were observed between other weather factors (maximum or minimum temperature, RH, precipitation, BP) and hip pain exacerbations [51]. The findings reinforce the idea that temperature changes play a role in exacerbating pain in individuals with hip OA, further highlighting the importance of understanding weather-related triggers for pain management strategies.

Moss et al. investigated the presence of hyperalgesia in response to pressure and cold in patients with knee osteoarthritis (OA) compared to healthy controls and found that OA subjects exhibited widespread hyperalgesia throughout the body in response to pressure and cold. This may indicate altered nociceptive function is not limited to the affected joint. Notably, there were no significant differences in heat pain thresholds between OA subjects and controls. These findings may suggest that pain in OA involves both local and central sensitization, and thus we must consider central factors in understanding and treating pain in OA [52].

The pathophysiology impacting the relationship between temperature and joint pain is likely multifactorial. Temperature may affect the compliance of periarticular structure, alter viscosity of synovial fluid, and affect capillary permeability indirectly affecting inflammatory mediators [53]. The theorized pathophysiology of this phenomena involves sensitivity of mechanoreceptors and thermoreceptors within affected joints [52–54]. Miller et al. posited that in OA, joint tissues generate and interact with cytokines and chemokines that promote joint degradation and directly stimulate local nociceptors [55]. Some also theorize that changes in temperature may induce vasoconstriction, leading to increased stiffness and discomfort [48, 49].

While the previous studies suggest that temperature may have a direct impact on patients’ symptoms, we must also consider how temperature impacts patients’ utilization of health care resources. A population-based, retrospective study with case-crossover design of 8,130 patients in Taiwan found that higher temperature range and humidity resulted in increased utilization of physical therapy and colder temperatures resulted in decreased utilization [56], suggesting that temperature may influence the rate at which patients seek medical attention.

Despite the available literature on the theorized pathophysiology of pain in osteoarthritis, there remain significant gaps in our understanding. Further studies aimed at elucidating these mechanisms are needed and may explore the roles of neurogenic inflammation, cytokine release, and changes in joint biomechanics in response to temperature fluctuations. Longitudinal studies examining OA patients may provide additional insight into the long-term effects of weather and temperature on disease progression and pain severity. Investigating potential interventions to mitigate the effects of temperature on OA pain also represents a promising area of research. This could include exploring the efficacy of thermal therapy, such as heat packs or cold compresses, in providing symptomatic relief for OA patients during adverse weather conditions.

## Conclusion

Chronic pain is a complex condition influenced by a variety of factors intrinsic and extrinsic to the patient. Environmental factors appear to play a significant role in pain exacerbations without much research to support this relatively ubiquitous patient experience. It has been postulated that changes in ambient temperature can impact the release of endogenous opioids, affect ion channels on primary afferent nerves and local cytokine release, as well as alter overall nerve function.

There is emerging evidence detailing the effect of ambient temperature on various chronic pain conditions such as FM, CRPS, MS and OA. Studies have shown that both decreases and increases in ambient temperatures can both exaggerate and generate pain in patients with FM. There are also studies detailing that patients with fibromyalgia have a narrower window for their temperature induced pain thresholds, emphasizing the role of temperature in this disease state. In MS the Uhthoff phenomenon is a well described exacerbation of neurologic symptoms, including pain, when individuals experience an increase in core body temperature. There is data, albeit limited, suggesting patients with CRPS also experience intensified pain in the setting of warm/hot weather. Lastly, there is some evidence supporting a variety of weather factors that affect pain in patients with OA, including temperature and other weather measurements.

Ambient temperature appears to contribute significantly to each of these disease states. More research needs to be done to elucidate the specific mechanisms underlying temperature-related pain exacerbations, explore the long-term effects of weather and temperature, and explore potential interventions to mitigate these effects in each of these conditions. Further, it has become clear that understanding any modifiable environmental factor may be important in guiding clinicians’ approach to care in these patients, as well as other chronic pain patients. Understanding the relationship between ambient temperature and pain is crucial for developing more effective pain management strategies for individuals with these conditions.

## Data Availability

No datasets were generated or analysed during the current study.
